# Symmetric Molecular Dynamics

**DOI:** 10.1021/acs.jctc.2c00401

**Published:** 2022-06-14

**Authors:** Sam Cox, Andrew D. White

**Affiliations:** Department of Chemical Engineering, University of Rochester, Rochester, New York 14627, United States

## Abstract

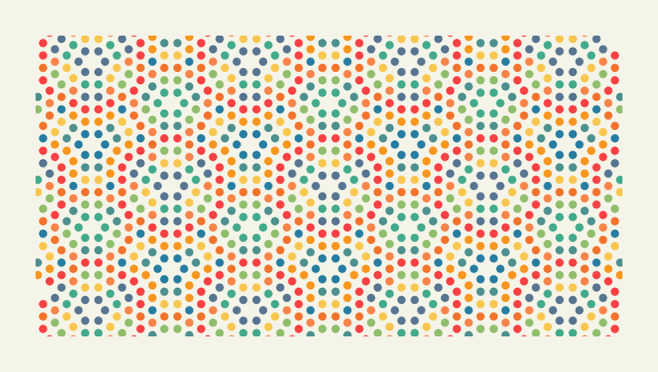

We derive a formulation of molecular dynamics that generates only symmetric configurations. We implement it for all 2D planar and 3D space groups. An atlas of 2D Lennard-Jones crystals under all planar groups is created with symmetric molecular dynamics.

Molecular dynamics has long been proposed as a method for predicting or understanding crystal structures.^1^ However, any practitioner will confess it is near impossible to observe point group symmetries in molecular dynamics. Here, we derive a constraint formulation of molecular dynamics where the symmetry group is an *input*. There is a finite number of symmetry groups. We simply simulate under all symmetry groups to generate symmetric structures.

There are two key ideas to our formulation that correspond to the two components of a space group: the point group symmetry and the Bravais lattice. The point group symmetry is treated as a holonomic constraint. The constraint equation is a function of positions that is zero when the positions are symmetric. Holonomic constraints are a relatively solved problem, and we follow previous approaches.^2−7^ The Bravais lattice is a constraint on the simulation lattice vectors that ensures the point group will tile space. Namely, the Bravais lattice specifies the relative lattice vector magnitudes and directions. We ensure our simulations are consistent by working in an unconstrained lattice vector space that is mapped to the correct Bravais lattice via a precomputed tensor. This frees us to use any NPT method in the unconstrained lattice vector space while still matching the Bravais lattice.

The concept of directly simulating under a symmetry group is unknown to us. The closest examples are methods like symmetry *restraints*.^8^ These harmonic restraints generally keep the system close to symmetric, but unlike the method we propose here, no single configuration is actually symmetric. Symmetry has certainly been considered as a *measure* of molecular configurations. For example, Zabrodsky et al.^9^ proposed a continuous symmetry measure, which is used to quantify the symmetry of atoms. This has been used to directly optimize Lennard-Jones clusters with symmetry.^10^ Of course, the direct use of symmetry for crystal structure prediction with Monte Carlo is common,^11,12^ and generative models with explicitly included symmetry are common.^13^ There are no molecular dynamics methods though that can directly sample space groups, which would be useful for crystal structure prediction and modeling biological assemblies.^14^ Symmetric molecular dynamics may also be viewed as a special case of objective molecular dynamics, which is a general method that encompasses any infinite or finite periodic tiling of a simulation.^15,16^ Similarly, others have explored generalizing periodic boundary conditions to other tilings.^17−20^

Below we derive our equations of motion and discuss implementation details. To assess the method, we show that it conserves energy and is capable of working in arbitrary space groups. Then we demonstrate its use to enumerate crystal structures of the Lennard-Jones potential under all planar groups with NPT simulations.

## I Theory

### A Equation of Motion

Consider the dynamics of *N* indistinguishable particles in *D* dimensions under a Hamiltonian *H*(**p**(*t*), **q**(*t*)). We wish to constrain *H* so that **q**(*t*) is symmetric at all times. Symmetry is a property of **q**(*t*) and a specific symmetry group of position transformations *G*, like mirrors along the *x* axis. **q**(*t*) is point group symmetric if applying any element of the group results in no change to the positions (ignoring ordering of particles)
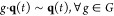
1where *g*· means applying the group element to each particle individually, ∼ means row equivalence, and *G* is a finite group. Group elements are represented as affine matrices in space and planar groups.

Eq 1 may hold trivially. For example, all particles are at the origin. Such special positions that are invariant to group elements are known as special Wyckoff positions.^21^ We remove this assumption in Section IIC, but for now additionally assume
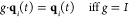
2where *I* is the identity transformation.

Assuming eqs 1 and 2 hold at *t* = 0, the particles can be partitioned into *N*/|*G*| = *n* group orbits. A group orbit is the set generated by applying all elements of group *G* to positions **q**_*i*_(*t*)
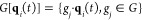
3

One member of all orbits will be **q**_*i*_(*t*) itself, because *G* contains the identity element. We can label the particles as *q*_*ij*_(*t*) where *i* indicates the orbit and *j* indicates the group element. In crystallography, the **q**_*i*0_ particles are called the asymmetric unit. We can satisfy eq 1 at all *t* by specifying the following holonomic constraint:
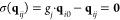
4

There are |*G*| – 1 of these constraints per group orbit, and each removes *D* degrees of freedom. This means the degrees of freedom of the dynamics is *D* × (*n* – 1). We can simulate dynamics under the holonomic constraints by simply only modeling the asymmetric unit—they are the generalized coordinates.^22^

Thus, our algorithm is to only integrate the asymmetric unit and explicitly consider the remaining (*N* – *n*) particles only when computing forces. This is similar to Dayal et al.^15^ Practically this is done by setting these constrained particles’ positions just before computing forces. Similar to work on periodic boundary conditions, these equations of motion may lead to linear momentum conservation problems.^23,24^

One feature of nearly all potentials used in molecular dynamics is that they are *G*-invariant, where *G* is any planar, space, or permutation group: *U*(*g*·**q**) = *U*(**q**). That makes the forces, *F*(**q**), *G*-equivariant
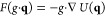
5because the potentials are composed of angles and distances, which are invariant to rotations, mirrors, and permutations.^25,26^ For a pairwise potential, we can use eqs 3 and 5 to rewrite the potential as
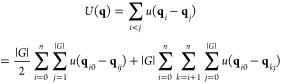
6where the |*G*| factor accounts for intragroup orbit interactions that are not explicitly computed. This translates an algorithm of an outer loop over the asymmetric unit and an inner loop over all particles.

### B Bravais Lattice

A space group consists of both a point group and a Bravais lattice. The Bravais lattice is specified with *D D*-dimensional unit cell vectors. Particles always remain in one cell among the lattice cells, which are called images. For example, we could simulate the “root” cell and its 26 neighboring cells in 3 dimensions. We follow the approach above and treat each image of the system with virtual particles while only integrating the root cell. This means all images of the system are explicit, and we can violate the minimum image convention. We were not signatories of the minimum image convention anyway. This approach allows the cell vectors to shrink well below the distance cutoff of the potential, provided we have enough virtual particles to populate past the cutoff of the asymmetric unit of the origin cell. You can simulate 3^*aD*^ images to allow the cells to shrink to at least 1/*a* the cutoff distance.

We need to convert between the fractional coordinates, which are used to tile the particles and apply the point group symmetry, to the Cartesian coordinates, which are used for integration and computed potentials. Given the box vectors in row-form *B*, we can transform between the representations via
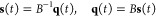
7where *s*(*t*) is the fraction of each lattice vector (i.e., fractional coordinates). Wrapping is trivial with fractional coordinates: *s*(*t*) fmod 1.0 will wrap the coordinates. All point group transformations are applied in **s**(*t*); however, a *B*^–1^ term should be added to eq 3 so that it operates on fractional coordinates.

Bravais lattices include more than just the usual cubic and triclinic lattices commonly seen in molecular dynamics barostats. To ensure the cell vectors are consistent with the Bravais lattice while changing box size, we define a tensor **L** of shape *D* × *D* × *D* × *D* that maps from a triclinic box vector to the proper Bravais lattice box vectors of the space group. For example, *L*_2011_ is the contribution to Bravais lattice vector 2’s *x* component from triclinic box vector 1’s *y* component. There are many choices that could be made for **L**. For example, to make a cubic Bravais lattice from a triclinic box vector, we require a single parameter *a* to define the three lattice vectors (*a*, 0, 0),(0, *a*, 0),(0, 0, *a*). We could set *a* by averaging all the vector lengths, averaging all vector components, or selecting *a* to be the first element of the first vector. Each of these choices gives a different **L**, and some have large null spaces. NPT is then accomplished via scaling Monte Carlo moves in the triclinic box vectors (*B*′) following Frenkel and Eppenga,^27^ and the proper Bravais lattice is computed via *B* = **L***B*′.

### C Wyckoff Positions

It is possible to have particles that violate eq 2 while still satisfying eq 1 if *q*_*i*0_ is in a special position called a Wyckoff position—like the origin.^21^ To perform constrained molecular dynamics of particle *q*_*i*0_(*t*) occupying a Wyckoff position, we define a subgroup *G*′ that contains the elements of *G* which do not leave *q*_*i*0_(*t*) invariant plus an identity group element. The identity of this subgroup is not the identity transform but instead a transform that projects from a general position into the Wyckoff position. For example, the Wyckoff position may be the vertical line *x* = 0, and the identity group element would be the transform *x*′ = 0, *y*′ = *y*. We will denote this group element as *P* to hint it is a projection.

The group orbit is similarly defined on the subgroup, and the other procedures above apply. However, *q*_*i*0_(*t*) must stay in a Wyckoff position at all times to satisfy eq 1. This can be accomplished via traditional constrained molecular dynamics of Lagrange multipliers.^28^ Omitting the indices on **q**_*i*0_(*t*), our holonomic constraint is
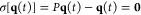
8and the force from the constraint will be
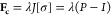
9where *J*[σ] is the Jacobian of σ with respect to constraint dimension and element of **q**(*t*). We can solve for λ by knowing that σ[**q**(*t* + Δ*t*)] = **0**
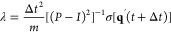
10where Δ*t* is the time step, *m* is the mass of the particle, and **q**′(*t* + Δ*t*) is **q**(*t*) integrated without the constraint force by Δ*t*. All terms are constant except σ[**q**′(*t* + Δ*t*)], which simplifies computation.

## II Methods

We use the BAOAB Langevin dynamics integrator described in refs (29 and 30). Eq 4 is applied during position updates, and eq 3 is applied before velocity updates (force computation). Degrees of freedom is computed from number of asymmetric unit particles and deducted degrees of freedom from Wyckoff position restraints. All simulations are Lennard-Jones potentials with cutoff 3.5 and in reduced units. NVE simulations are conducted with the velocity-verlet integrator. A time step of 0.005 and a Langvin γ of 0.1 were used for all simulations. Since images are explicit in our implementation, it is necessary to specify the number. We use an image radius of 2–meaning 3^2*D*^ images are simulated where *D* is the dimension. To generate starting configurations, points were randomly generated and filtered to fit into the space group asymmetric unit as specified by Aroyo.^31^ Point group generators and Wyckoff sites were taken from the Bilbao crystallography server.^32−34^

We define our results in reduced units, as defined in ref (35). Specifically, energy (ϵ) is fundamental, and τ is a derived unit of the form
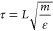
11where *L*, *m*, and ϵ are the fundamental units of length, mass, and energy, respectively.

## III Results

**Figure 1:**
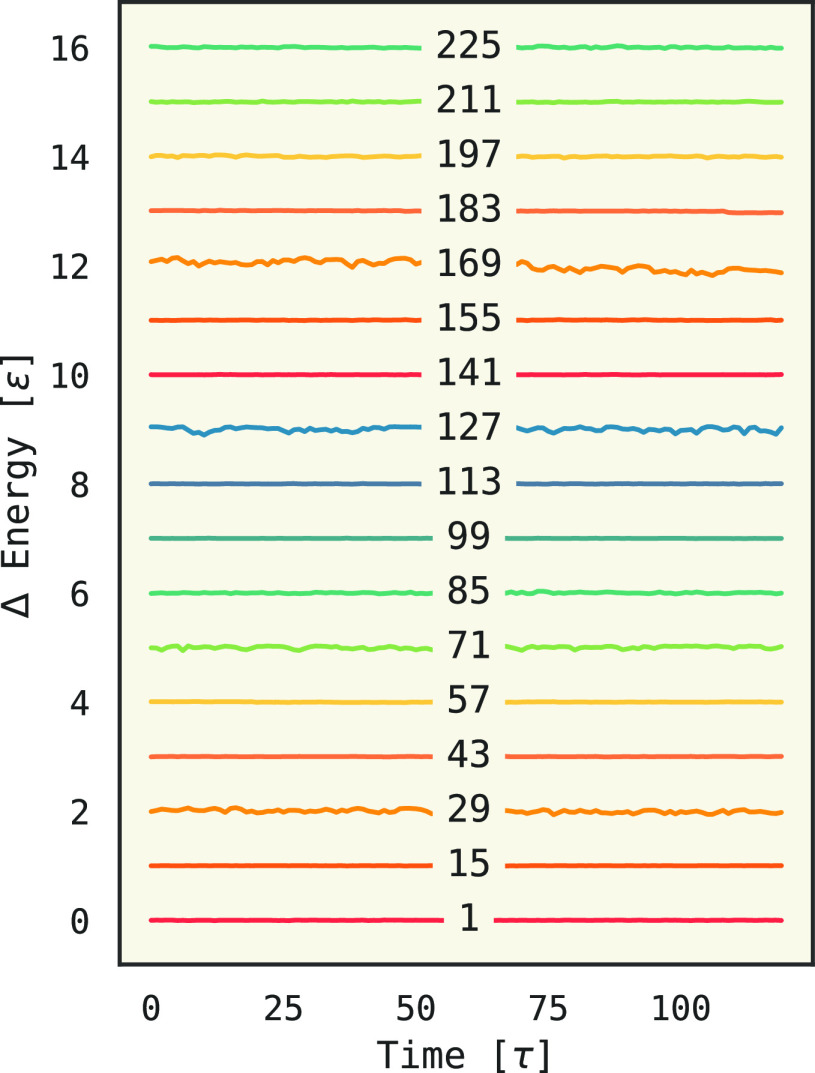
Total energy from NVE simulations under different symmetry groups in 3D. Groups are indicated with Hall numbers. Four particles are in the asymmetric unit, and the simulations are at a number density of 0.2 and a starting temperature of 0.5. The increase in fluctuations is because the unit cell (total particles) increases with the size of the symmetry group.

We first consider if our implementation conserves energy. Figure 1 shows the total energy of NVE simulations under a subset of space groups with 5 particles in the asymmetric unit. These were done at number densities of 0.2, with a starting temperature 0.5, and for 30k timesteps. The bottom trace (P1) has no symmetry constraints and shows good conservation. There are more fluctuations at other symmetry groups because there are more particles in their unit cells and thus higher energy fluctuations. For example, space group 127 (*P*4/*mbm*) has 80 particles in a unit cell when there are 5 in the asymmetric unit, meaning the interaction potential felt has more particles contributing to it.

**Figure 2:**
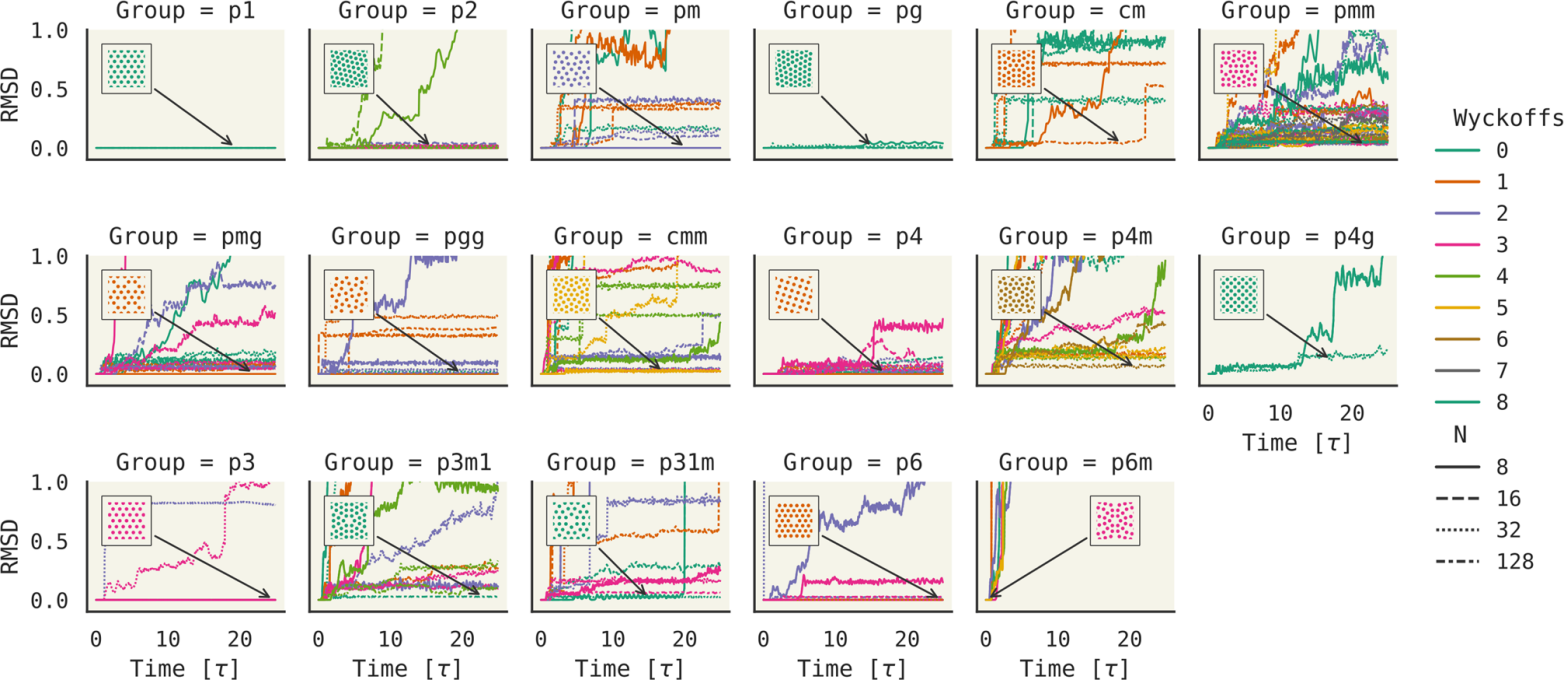
An atlas of 258 Lennard-Jones crystal structures. Subplots are broken down by planar group, and each line color/style indicates Wyckoff occupancy and unit cell particle count. Individual plots show RMSD from the starting configuration, which was constrained to match the planar group in subplot titles. RMSD was calculated during the NVE simulation of 5k steps with no symmetry constraints. A rise indicates change of configuration–meaning the starting configuration was not stable. Missing traces indicate either simulation diverged or number density did not exceed 0.5.

Figure 2 shows an enumeration of crystal structures under different symmetry groups for a 2D Lennard-Jones fluid. The structures are generated in 2 steps. First, we simulate under a symmetry group constraint in NPT (*P* = 0.25, *T* = 0.1) for 1 M steps. Next, we do a constrained equilibration under NVT for 100k steps at *T* = 0.05. This structure is then the proposed crystal structure for the given symmetry group. Figure 2 shows the root mean square deviation (RMSD) if the resultant structure is simulated under no symmetry constraint in NVE for 5k steps. The assumption is that if the structure does not collapse (RMSD rise), it is metastable. We indeed find that this protocol under no symmetry constraints (p1) gives the correct hexagonal packing.

To enumerate all planar groups in 2D, we simulate under each group, with 4 choices of particle number (in unit cell) and varying occupancy of Wyckoff sites. As expected, the planar groups with hexagonal Bravais lattices (or permit them) have stable structures: p1, p2, pg, p3, p6, and cmm.^36^ Some unusual stable structures are seen without hexagonal close-packing like p4m and p3m1. Metastable structures like these would be nearly impossible to generate without symmetry constraints in molecular dynamics. We find some symmetries have no metastable structures: p6m and most square close-packing (cm, cmm, p4g). Interestingly, voids seem to be the way to stabilize these close-packing structures like in pmg.

## IV Discussion

Here is our advice on implementing symmetric molecular dynamics in a modern molecular dynamics engine. The asymmetric unit should be integrated as usual. Make the nonasymmetric unit particles (images) be ghost particles; ghost particles are nonintegrated particles used in force-field calculations. The ghost particles’ positions should be set using affine matrices defining the group transformations in fractional coordinates. These matrices can be obtained from our library or crystallography tables.

Pressure computed for the asymmetric unit is not meaningful, and NPT should be done using the algorithm described above that does not require computing pressure from a virial. The lattice vectors may be stored separately than the usual lattice vectors and are only used to set the ghost particle positions. The tensor transforms can be loaded from our library. Periodic boundary conditions should be disabled entirely if doing NPT.

The constraints for Wyckoff sites are implemented as Lagrange multiplier constraints. The terms can be computed analytically at each step using eq 10.

## V Conclusions

We have formulated a symmetric molecular dynamics algorithm and implemented it. Results show that it can do NPT to enumerate metastable crystal structures. A reference implementation is available at https://github.com/whitead/symd.

## References

[ref1] Parrinello M.; Rahman A. Polymorphic transitions in single crystals: A new molecular dynamics method. J. Appl. Phys. 1981, 52, 7182 10.1063/1.328693.

[ref2] Ryckaert J.-P.; Ciccotti G.; Berendsen H. J. Numerical integration of the cartesian equations of motion of a system with constraints: molecular dynamics of nalkanes. J. Comput. Phys. 1977, 23, 327 10.1016/0021-9991(77)90098-5.

[ref3] Andersen H. C. Rattle: A “oevelocity” version of the shake algorithm for molecular dynamics calculations. J. Comput. Phys. 1983, 52 (1), 24–34. 10.1016/0021-9991(83)90014-1.

[ref4] Tobias D. J.; Brooks C. L. III Molecular dynamics with internal coordinate constraints. J. Chem. Phys. 1988, 89, 5115 10.1063/1.455654.

[ref5] Ryckaert J.-P. Special geometrical constraints in the molecular dynamics of chain molecules. Mol. Phys. 1985, 55, 549 10.1080/00268978500101531.

[ref6] Edberg R.; Evans D. J.; Morriss G. Constrained molecular dynamics: Simulations of liquid alkanes with a new algorithm. J. Chem. Phys. 1986, 84, 6933 10.1063/1.450613.

[ref7] Ciccotti G.; Ryckaert J.-P. Molecular dynamics simulation of rigid molecules. Computer Physics Reports 1986, 4, 346 10.1016/0167-7977(86)90022-5.

[ref8] Anishkin A.; Milac A. L.; Guy H. R. Symmetryrestrained molecular dynamics simulations improve homology models of potassium channels. Proteins: Struct., Funct., Bioinf. 2010, 78, 932 10.1002/prot.22618. PMC2811770 19902533

[ref9] Zabrodsky H.; Peleg S.; Avnir D. Continuous symmetry measures. J. Am. Chem. Soc. 1992, 114, 7843 10.1021/ja00046a033.

[ref10] Oakley M. T.; Johnston R. L.; Wales D. J. Symmetrisation schemes for global optimization of atomic clusters. Phys. Chem. Chem. Phys. 2013, 15, 3965 10.1039/c3cp44332a. 23389762

[ref11] van Eijck B. P.; Kroon J. Upack program package for crystal structure prediction: Force fields and crystal structure generation for small carbohydrate molecules. Journal of computational chemistry 1999, 20, 799 10.1002/(SICI)1096-987X(199906)20:8<799::AID-JCC6>3.0.CO;2-Z. 35619460

[ref12] Fredericks S.; Parrish K.; Sayre D.; Zhu Q. Pyxtal: A python library for crystal structure generation and symmetry analysis. Comput. Phys. Commun. 2021, 261, 107810 10.1016/j.cpc.2020.107810.

[ref13] Xie T.; Fu X.; Ganea O.-E.; Barzilay R.; Jaakkola T. Crystal diffusion variational autoencoder for periodic material generation. 2021, arXiv:2110.06197. arXiv preprint. https://arxiv.org/abs/2110.06197 (accessed 2022-06-10).

[ref14] Cannon K. A.; Ochoa J. M.; Yeates T. O. High-symmetry protein assemblies: patterns and emerging applications. Curr. Opin. Struct. Biol. 2019, 55, 77 10.1016/j.sbi.2019.03.008. 31005680

[ref15] Dayal K.; James R. D. Nonequilibrium molecular dynamics for bulk materials and nanostructures. Journal of the Mechanics and Physics of Solids 2010, 58, 145 10.1016/j.jmps.2009.10.008.

[ref16] Xu H.; Drozdov G.; Hourahine B.; Park J. G.; Sweat R.; Frauenheim T.; Dumitrică T. Collapsed carbon nanotubes: From nano to mesoscale via density functional theory-based tight-binding objective molecular modeling. Carbon 2019, 143, 786 10.1016/j.carbon.2018.11.068.

[ref17] Hansson T.; Oostenbrink C.; van Gunsteren W. Molecular dynamics simulations. Curr. Opin. Struct. Biol. 2002, 12, 190 10.1016/S0959-440X(02)00308-1. 11959496

[ref18] Denton R.; Hu Y. Symmetry boundary conditions. J. Comput. Phys. 2009, 228, 4823 10.1016/j.jcp.2009.03.033.

[ref19] Wagner G. J.; Karpov E. G.; Liu W. K. Molecular dynamics boundary conditions for regular crystal lattices. Computer Methods in Applied Mechanics and Engineering 2004, 193, 1579 10.1016/j.cma.2003.12.012.

[ref20] Roy A.; Post C. B. Microscopic symmetry imposed by rotational symmetry boundary conditions in molecular dynamics simulation. J. Chem. Theory Comput. 2011, 7, 3346 10.1021/ct2000843. 22096451 PMC3215146

[ref21] Wyckoff R. W. G. The Analytical Expression of the Results of the Theory of Space-groups; Carnegie Institution of Washington: 1922; Vol. 318.

[ref22] You can also arrive at this solution by constraining the Cartesian coordinates and finding Lagrange multipliers.

[ref23] Shirts R. B.; Burt S. R.; Johnson A. M. Periodic boundary condition induced breakdown of the equipartition principle and other kinetic effects of finite sample size in classical hard-sphere molecular dynamics simulation. J. Chem. Phys. 2006, 125, 164102 10.1063/1.2359432. 17092058

[ref24] Kuzkin V. A. On angular momentum balance for particle systems with periodic boundary conditions. ZAMM-Journal of Applied Mathematics and Mechanics/ Zeitschrift für Angewandte Mathematik und Mechanik 2015, 95, 1290 10.1002/zamm.201400045.

[ref25] Musil F.; Grisafi A.; Bart′ok A. P.; Ortner C.; Cs′anyi G.; Ceriotti M. Physics-inspired structural representations for molecules and materials. Chem. Rev. 2021, 121, 9759 10.1021/acs.chemrev.1c00021. 34310133

[ref26] White A. Deep learning for molecules and materials; 2021. https://whitead.github.io/dmol-book/ (accessed 2022-06-01). 10.33011/livecoms.3.1.1499 PMC10727448 38111390

[ref27] Frenkel D.; Eppenga R. Monte carlo study of the isotropic-nematic transition in a fluid of thin hard disks. Physical review letters 1982, 49, 1089 10.1103/PhysRevLett.49.1089.

[ref28] Miyamoto S.; Kollman P. A. Settle: An analytical version of the shake and rattle algorithm for rigid water models. Journal of computational chemistry 1992, 13, 952 10.1002/jcc.540130805.

[ref29] Leimkuhler B.; Matthews C. Robust and efficient configurational molecular sampling via langevin dynamics, The. J. Chem. Phys. 2013, 138, 174102 10.1063/1.4802990. 23656109

[ref30] Leimkuhler B.; Matthews C. Efficient molecular dynamics using geodesic integration and solvent-solute splitting. Proceedings of the Royal Society A: Mathematical, Physical and Engineering Sciences 2016, 472, 20160138 10.1098/rspa.2016.0138. PMC4893190 27279779

[ref31] Aroyo M. I. International Tables for Crystallography; Wiley Online Library: 2013.

[ref32] Aroyo M. I.; Perez-Mato J.; Orobengoa D.; Tasci E.; de la Flor G.; Kirov A. Crystallography online: Bilbao crystallographic server. Bulg. Chem. Commun. 2011, 43, 183.

[ref33] Aroyo M. I.; Perez-Mato J. M.; Capillas C.; Kroumova E.; Ivantchev S.; Madariaga G.; Kirov A.; Wondratschek H. Bilbao crystallographic server: I. databases and crystallographic computing programs. Zeitschrift für Kristallographie-Crystalline Materials 2006, 221, 15 10.1524/zkri.2006.221.1.15.

[ref34] Aroyo M. I.; Kirov A.; Capillas C.; Perez-Mato J.; Wondratschek H. Bilbao crystallographic server. ii. representations of crystallographic point groups and space groups. Acta Crystallographica Section A: Foundations of Crystallography 2006, 62, 115 10.1107/S0108767305040286. 16489249

[ref35] Frenkel D.; Smit B.; Ratner M. A. Understanding molecular simulation: from algorithms to applications; Academic Press: San Diego, 1996; Vol. 2.

[ref36] This depends on Wyckoff site occupancy too.

